# Transcriptome Profiling Reveals Stage-Specific Regulation of Lipid Metabolism in Orbital Fat of Bighead Carp (*Hypophthalmichthys nobilis*)

**DOI:** 10.3390/ani15172602

**Published:** 2025-09-04

**Authors:** Junru Wang, Qi Lei, Jun Liu, Zhiruo Sun, Xiaomu Yu, Xusheng Guo, Jingou Tong

**Affiliations:** 1 School of Fisheries, Xinyang Agriculture and Forestry University, Xinyang 464000, China; jrwang1021@163.com (J.W.); aqualeiq@163.com (Q.L.); liujunhnsd@126.com (J.L.); 15664013605@163.com (Z.S.); 2 Fishery Biological Engineering Technology Research Center of Henan Province, Xinyang 464000, China; 3 State Key Laboratory of Freshwater Ecology and Biotechnology, Institute of Hydrobiology, Academy of Seed Design, Chinese Academy of Sciences, Wuhan 430072, China; xmyu@ihb.ac.cn

**Keywords:** bighead carp, orbital fat, transcriptome, differentially expressed genes (DEGs)

## Abstract

The orbital fat of bighead carp (*Hypophthalmichthys nobilis*) represents a structural fat deposit located posterior to the eyes and constitutes an important edible component of the head region. Nevertheless, molecular mechanisms governing lipid accumulation during ontogenetic development remain insufficiently characterized. Here, we performed RNA-Seq on orbital fat tissues from 6-month-old (juvenile) and 18-month-old (market-size) bighead carp. A total of 1042 DEGs were identified, with 807 up-regulated and 235 down-regulated in the 6-month-old stage. Functional enrichment revealed key pathways including fatty acid metabolism, PPAR signaling, and glycolysis/gluconeogenesis. qRT-PCR validation confirmed RNA-Seq reliability. Notably, the differential expression patterns of genes such as *cpt1a*, *cpt1b*, *slc27a1*, *fads2*, and *scd* suggest their association with an elevated capacity for lipid synthesis in the orbital fat of 18-month-old bighead carp. This study presents the first transcriptome analysis of orbital fat development in a freshwater fish, offering insights into the genetic improvement of head meat quality traits and growth in bighead carp head.

## Simple Summary

The head of bighead carp, particularly the flavorful orbital fat behind the eyes, is a prized culinary component. Understanding how fat accumulates in this specific tissue as the fish grows is crucial for improving its quality. However, the underlying molecular mechanisms remain poorly understood. To address this, we compared the gene activity (transcriptome) in orbital fat between juvenile (6-month-old) and market-size (18-month-old) bighead carp. Key biological pathways involved in fat production and breakdown, such as fatty acid metabolism and PPAR signaling, showed significant changes. Importantly, genes like *cpt1a*, *cpt1b*, *slc27a1*, *fads2*, and *scd* were found to be linked to increased fat synthesis capacity in the market-size fish. This study provides the first detailed genetic blueprint of orbital fat development in the freshwater fish. These findings offer valuable insights and potential gene targets for future breeding programs aimed at enhancing the growth and nutritional quality of the bighead carp head for consumers.

## 1. Introduction

Adipose tissue has a significant effect on meat quality and animal productivity [1]. Fat accumulation is influenced by age, genetics, and nutrition, with metabolic processes within adipose tissue directly governing fat deposition [2]. Notably, adipose tissue comprises adipocytes in addition to numerous other cell types, including endothelial cells, immune cells, fibroblasts, preadipocytes, and stem cells. The differentiation of adipocytes is a vital process that dictates the level of lipid accumulation within the tissue, and this differentiation is managed by an intricate network of molecular events [3].

Teleost fish exhibit lipid accumulation across a wide range of anatomical regions including visceral organs, liver, subcutaneous tissue, both red and white muscle tissue, brain, pancreas, esophagus, jaw, cranial skeleton, and caudal fin. This distribution varies based on species, nutritional status, life stage, and physiological condition [4]. Analysis of age-specific lipid and fatty acid composition in Atlantic salmon juveniles (at 0+, 1+, and 2+ years) revealed that the regulatory stability of critical functions in developing organisms is maintained through structural changes in the lipid system [5]. This suggests that when studying developmental regulation in different age stages of fish, in addition to muscles and bones, the regulation of the lipid system is also worthy of attention.

Fish have long been recognized as the most important source of long-chain polyunsaturated fatty acids (LC-PUFA) for human consumption, as well as a feed component for farmed fish and other food animals [6,7]. Unlike marine fish, freshwater teleosts possess the ability to desaturate and elongate 18C fatty acids into 20–22C LC-PUFAs. It is noteworthy that in the biosynthesis of LC-PUFA, the Δ6 desaturase (FADS2), which catalyzes the first desaturation step, plays a crucial role and is often used as an indicator of the LC-PUFA synthesis capacity in fish [8]. The influence of dietary fatty acid composition on fish growth, lipid peroxidation, mitochondrial lipids, and gene expression demonstrates age-dependent variations. A study investigating the effects of dietary fatty acids on mitochondrial phospholipid composition, oxidative status, and mitochondrial gene expression in zebrafish of varying ages revealed that younger fish are in a growth phase, whereas older fish are not. As a result, older fish exhibit a decreased susceptibility to lipid peroxidation [9]. These insights suggest that the ability for lipid regulation in fish undergoes modifications related to age.

Compared to other tissues, fish heads may contain higher levels of lipids, including PUFAs, excluding the visceral organs [10]. In particular, orbital fat, which serves a protective role around the optic nerve, has been identified in marine species like tuna and skipjack as a significant reservoir of highly unsaturated fats, with DHA comprising a substantial proportion [11,12]. This highlights the potential of fish head-derived lipids—especially from orbital fat—as a valuable nutritional source. The regulation of lipid metabolism and deposition is tightly controlled at the molecular level, with gene expression patterns in adipose tissue playing a key role in mediating responses to environmental and developmental cues. Transcriptomic analysis provides a powerful tool for rapidly capturing the sequences and expression levels of nearly all transcripts within tissues under specific conditions and has been widely applied to study various traits in fish [13]. The gene expression patterns in adipose tissue are particularly important, as they help link environmental variations to energy balance and maturation timing through genetic mechanisms that integrate lipid metabolism, seasonality, and sexual maturation [14]. For example, a study investigating the effects of glycyrrhizic acid (GA) supplementation in blunt snout bream juveniles revealed that 0.3 mg/kg GA significantly improved growth performance and lipid metabolism [15]. Similarly, transcriptomic analysis of subcutaneous adipose tissue in yellow drum fed a diet containing optimal n-3 LC-PUFAs (9.8 g/kg) showed that differentially expressed genes were primarily enriched in peroxisome and fatty acid biosynthesis pathways [16].

Bighead carp (*Hypophthalmichthys nobilis*) is a major freshwater aquaculture species in China and one of the traditional “Four Major Domestic Fish”. Unlike many other fish species, the head of bighead carp is highly prized by consumers, often exceeding the economic value of the body meat. Notably, the brain tissue of bighead carp is rich in polyunsaturated fatty acids (PUFAs). Interestingly, the ratio of EPA + DHA to total fatty acids in the total lipid (TL) and triglyceride (TG) fractions of bighead carp head shows no significant difference compared to that of Atlantic salmon—a representative marine fish [17]. In particular, the orbital fat and the associated extraocular muscles are regarded as the most flavorful parts of the head and are key constituents in gourmet dishes. In this study, we conducted transcriptome sequencing of orbital fat tissues from bighead carp at two critical developmental stages: juvenile (6 months old) and market-size (18 months old). By comparing gene expression profiles, with emphasis on lipid synthesis pathways, we aimed to elucidate the molecular mechanisms governing the growth and development of orbital fat, as well as the genetic regulation of fat deposition and metabolism within the head region. The acquisition of relevant pathways and important candidate genes for fat regulation will provide a theoretical basis for the genetic improvement and commercial development of bighead carp head growth and nutritional quality traits.

## 2. Materials and Methods

### 2.1. Experimental Material

The materials used in this study were obtained from a mixed-family fish strain cultivated at Zhangdu Lake Fishery in Wuhan, China. The fish were reared in ponds and fed the same commercial diet (Tongwei 131, Tongwei Co., Ltd., Chengdu, China; protein content ≥ 30%) throughout the experimental period under standardized farming conditions. Samples were collected at 6 months post-hatch (M6) (body weight: 512 ± 4.25 g) and at 18 months (M18) (body weight: 2867 ± 36.54 g). For each time point, three individual fish (mixed sex) were used, with each fish representing one biological replicate. After anesthesia with ethyl 3-amino-benzoic acid methyl sulfonate (MS-222) (Sigma-Aldrich, St. Louis, MO, USA), the orbital adipose tissue was rapidly dissected and flash-frozen in liquid nitrogen. The orbital adipose tissues from both eyes of each fish were pooled into one tube as a single sample and stored at −80 °C in an ultra-low temperature freezer.

### 2.2. RNA Extraction and Quality Evaluation

Total RNA was isolated from all samples with TRIzol reagent (Invitrogen, Carlsbad, CA, USA), followed by DNase I treatment to eliminate genomic DNA contamination. RNA quality was initially evaluated by 1% agarose gel electrophoresis to detect potential degradation or impurities. The purity and concentration of RNA were measured using a Nanodrop2000 spectrophotometer (Thermo Scientific, Waltham, MA, USA), while RNA integrity was further confirmed with an Agilent 2100 Bioanalyzer System (Agilent Technologies, Santa Clara, CA, USA) employing the Agilent RNA 6000 Nano Kit (Agilent Technologies, Santa Clara, CA, USA). Only high-quality RNA samples that passed these quality controls were proceeded to Illumina library preparation. Library preparation and next-generation sequencing were performed by Biomarker Technologies Co., Ltd. (Beijing, China). The final libraries were sequenced on an Illumina NovaSeq 6000 (San Diego, CA, USA) platform to generate 150 bp paired-end reads.

### 2.3. RNA Sequencing and Data Analysis

Raw sequencing reads were processed to remove adapters and low-quality sequences, yielding high-quality clean reads for all subsequent analyses. The clean reads were then aligned to the bighead carp reference genome (NCBI BioProject accession: PRJNA1090045) using HISAT2 (2.2.1). The resulting SAM files were converted to BAM files, sorted, and indexed using SAMtools (v1.12). Gene-level read counts were generated from the uniquely mapped reads using featureCounts (from Subread v2.0.3). Differential gene expression analysis was performed on the raw count matrix using the DESeq2 (v1.30.1). Simultaneously, the expression levels of genes were also calculated as Fragments Per Kilobase of exon per Million mapped fragments (FPKM) for downstream visualization and interpretation.

The resulting *p*-values were adjusted for multiple testing using the Benjamini–Hochberg method to control the false discovery rate (FDR). Genes with an FDR ≤ 0.01 and an absolute fold change ≥ 2 were defined as significantly differentially expressed. GO and KEGG enrichment results using the clusterProfiler R package (v4.4.1) with the Benjamini–Hochberg (BH) correction applied across all terms within the three GO domains (Biological Process, Cellular Component, Molecular Function) and KEGG pathways simultaneously. The protein–protein interaction network was constructed using the GeneMANIA plugin (version 3.6.0) in Cytoscape (version 3.9.1). Due to the lack of extensive interaction data specifically for bighead carp, the analysis was performed using the *Homo sapiens* reference database available within GeneMANia, which integrates interaction data from multiple sources including physical interactions, genetic interactions, pathways, co-expression, co-localization, and protein domain similarity. A permutation test (*n* = 1000) was conducted within the GeneMANIA framework to assess the statistical significance of the observed network connectivity. The resulting network is considered to represent functional associations that are likely to be conserved across vertebrates.

### 2.4. Validation of RNA-Seq Results by Quantitative Real-Time PCR (qRT-PCR)

To validate the RNA-seq results, ten differentially expressed genes (DEGs) were randomly selected for quantitative real-time PCR (qRT-PCR) analysis, with β-actin serving as the internal reference gene. The ten differentially expressed genes (DEGs) selected for qRT-PCR validation were chosen randomly based on the following criteria to ensure a comprehensive evaluation of the RNA-seq data: (1) a wide range of fold-change values (both up- and down-regulated in the M6); (2) representation of key significantly enriched KEGG pathways, namely fatty acid metabolism, Tight junction, PPAR signaling pathway, and glycolysis/gluconeogenesis. Total RNA used for qRT-PCR was obtained from the same biological specimens as those utilized for transcriptome sequencing. cDNA was reverse-transcribed from 1 μg of total RNA per sample using the PrimeScript™ RT kit (TaKaRa, Dalian, China). Gene-specific primers were designed with Primer 5 software, and their sequences are provided in Table 1. All qRT-PCR reactions were conducted on a StepOne™ Real-Time PCR System (Applied Biosystems, Carlsbad, CA, USA). Each 20 μL qRT-PCR reaction consisted of 10 μL of SYBR Green Master Mix, 0.6 μL of each primer (2 μmol/L), 2 μL of cDNA template (corresponding to 1000 ng total RNA), and 6.8 μL of nuclease-free water. The thermal cycling conditions were as follows: initial denaturation at 95 °C for 10 min, followed by 40 cycles of 95 °C for 15 s, 60 °C for 30 s, and 72 °C for 45 s. Each sample was analyzed in three biological and three technical replicates. Gene expression levels were calculated using the 2−^ΔΔCT^ method normalized to β-actin [18]. The correlation between the RNA-seq and qRT-PCR results was assessed by linear regression analysis.

## 3. Results

### 3.1. Transcriptome Sequencing

**Figure 1:**
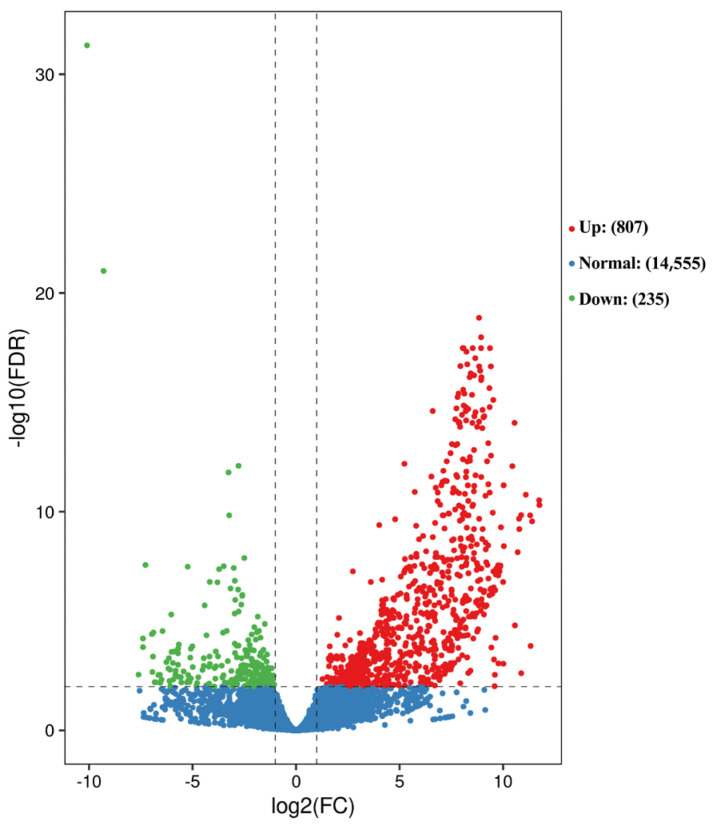
Patterns of differentially expressed genes (DEGs). Note: Red or green dots represent differentially expressed genes, blue dots represent non differentially expressed genes.

RNA-Seq was performed on orbital fat from bighead carp at two developmental stages (M6, M18), with three biological replicates per stage (total n = 6). A total of 40.24 Gb of clean data was obtained, averaging 6.71 Gb per sample. The Q30 scores for all samples exceeded 91.73%, and GC content ranged from 45.37% to 48.38% (Table A1). All sequencing data were uploaded to the Sequence Reading Archive (SRA) of the National Center for Biotechnology Information (NCBI; accession number: PRJNA1284284). In comparison with M18 vs. M6, 1042 DEGs were identified: 807 were significantly up-regulated in M6, and 235 were significantly down-regulated in M6 (Figure 1).

### 3.2. GO and KEGG Enrichment Analysis of Differentially Expressed Genes

**Figure 2:**
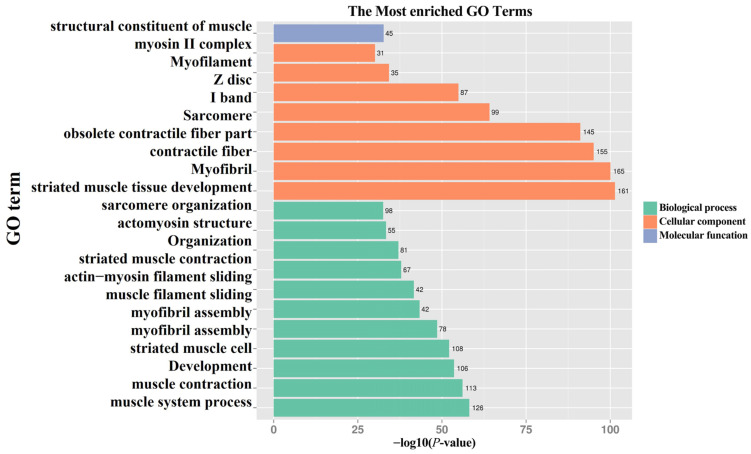
Top 20 GO classifications for enrichment analysis of DEGs at two stages of orbital fat of bighead carp. Note: The numbers right the bars indicate the count of genes enriched in each term.

To deeply explore the biological significance of DEGs in the two growth stages of orbital fat, we ranked the enriched terms based on ascending *p*-value and mainly analyzed the top 20 GO entries (Figure 2). Functional enrichment analysis shows that DEGs in two periods were mainly clustered in cellular components and biological processes, with fewer DEGs clustered in molecular function. In cellular components, DEGs mainly include myofibril (GO:0030016), contractile fiber (GO:0043292), and sarcomere (GO:0030017), etc. In biological process, DEGs mainly include muscle system process (GO:0003012), muscle contraction (GO:0006936), muscle cell development (GO:0055001), etc.; In molecular function, DEGs are mainly structural constituent of muscle (GO:0008307). GO enrichment analysis was performed using the clusterProfiler R package (v4.4.1), with Benjamini–Hochberg (BH) correction applied simultaneously across all terms from the three Gene Ontology (GO) domains (Biological Process, Cellular Component, and Molecular Function). The results are presented in Figure A2.

**Figure 3:**
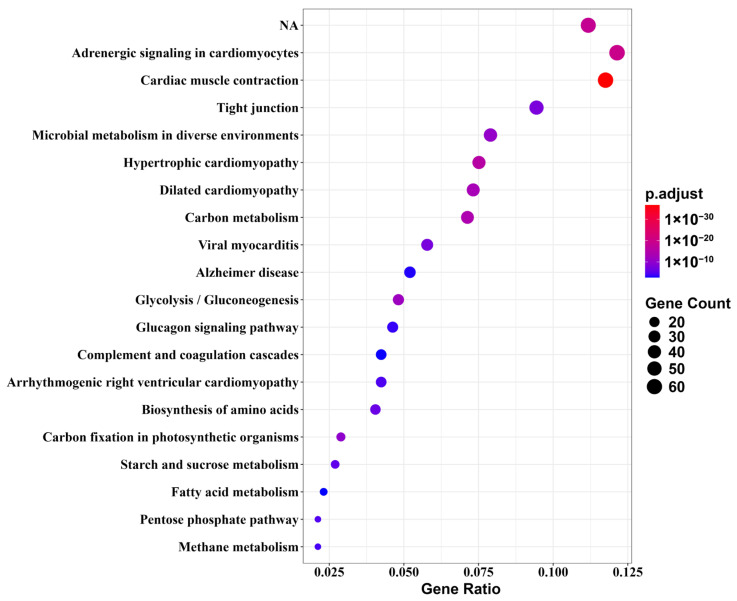
Top 20 enriched KEGG pathways of DEGs in orbital fat of bighead carp. The color of the circles represents the significance of enrichment (−log_10_(adjusted *p*-values)), and the size of the circles represents the number of genes enriched in each pathway.

KEGG enrichment analysis showed that the pathways that were significantly enriched in the comparison between M6 and M18 were mainly Cardiac muscle contraction, Adrenergic signaling in cardiomyocytes, carbon metabolism, Glycolysis/Gluconeogenesis, fatty acid metabolism, PPAR signaling pathway, etc. (Figure 3).

### 3.3. Important Candidate Genes Regulating the Growth and Development of Adipose Tissue in the Bighead’s Eye Socket

**Figure 4:**
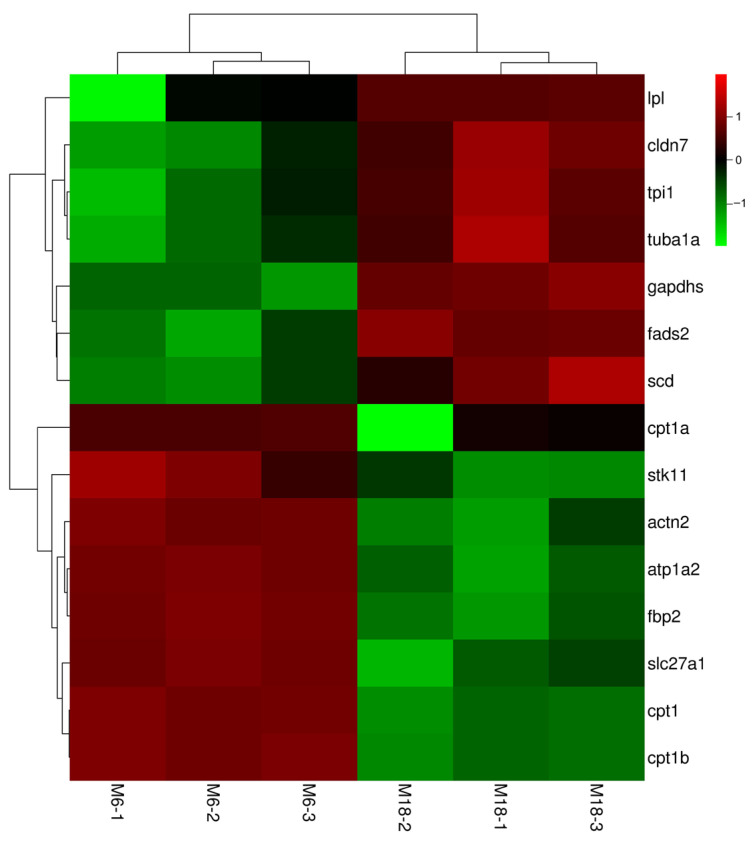
Heat map of 15 important DEGs expression profiles for the two growth stages. Gene abbreviations: lipoprotein lipase (*lpl*), claudin 7 (*cldn7*), triosephosphate isomerase 1 (*tpi1*), tubulin alpha 1a (*tuba1a*), glyceraldehyde-3-phosphate dehydrogenase, spermatogenic (*gapdhs*), fatty acid desaturase 2 (*fads2*), stearoyl-CoA desaturase (*scd*), carnitine palmitoyltransferase 1A (*cpt1a*), serine/threonine kinase 11 (*stk11*), actinin alpha 2 (*actn2*), ATPase Na+/K+ transporting subunit alpha 2 (*atp1a2*), fructose-bisphosphatase 2 (*fbp2*), solute carrier family 27 member 1 (*slc27a1*), carnitine palmitoyl transferase I (*cpt1*), carnitine palmitoyltransferase 1B (*cpt1 b*).

**Figure 5:**
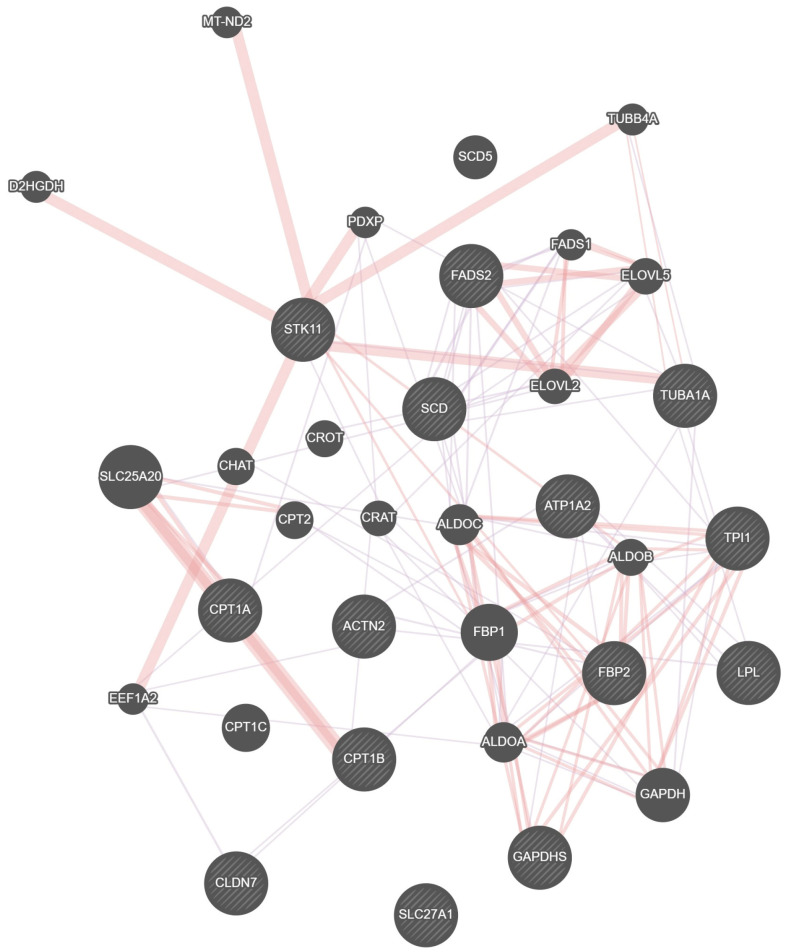
Correlation network of key genes. Diagonally filled circular nodes indicate key genes associated with early growth regulation in orbital fat. Circular nodes filled with solid colors indicate genes that interact with key genes. Interactions between different genes are indicated by different colored lines. Red lines indicate predicted interactions. Purple lines indicate co-expression interactions.

Through KEGG pathway enrichment analysis, six key pathways and associated candidate genes were identified. These genes may play important roles in bighead carp orbital fat growth and development and fat regulation (Figure 3; Table 2). We analyzed important DEGs in key pathways that showed a high frequency of involvement (Figure 4) and used Gene MANIA to construct correlation networks out of these 15 key DEGs (Figure 5). Up-regulated DEGs were primarily enriched in pathways such as Cardiac muscle contraction and Adrenergic signaling in cardiomyocytes. The pathways Fatty acid metabolism, Tight junction, PPAR signaling pathway, and Glycolysis/Gluconeogenesis contained both up- and down-regulated DEGs. Among the DEGs enriched in key pathways, 23 genes exhibited higher expression at M6 (i.e., ryanodine receptor 3 (*ryr3*), ATPase Na+/K+ transporting subunit alpha 2 (*atp1a2*), actin alpha cardiac muscle 1 (*actc1*), tropomyosin 2 (*tpm2*), hydroxy acyl-CoA dehydrogenase (*hadh*), carnitine palmitoyl transferase 1B (*cpt1b*), acyl-CoA synthetase long chain family member 1 (*acsl1*) and fructose-bisphosphates 2 (*fbp2*), among others). Conversely, 18 genes were down-regulated in the M6 group, such as fatty acid desaturase 2 (*fads2*), tubulin alpha 1a (*tuba1a*), apolipoprotein A1 (*apoa1*), fructose-bisphosphates 1 (*fbp1*), hexokinase 2 (*hk2*), enolase 1 (*eno1*), and triosephosphate isomerase 1 (*tpi1*) are among the genes associated with lipid anabolism.

### 3.4. Validation of RNA-Seq Results by qPCR

**Figure 6:**
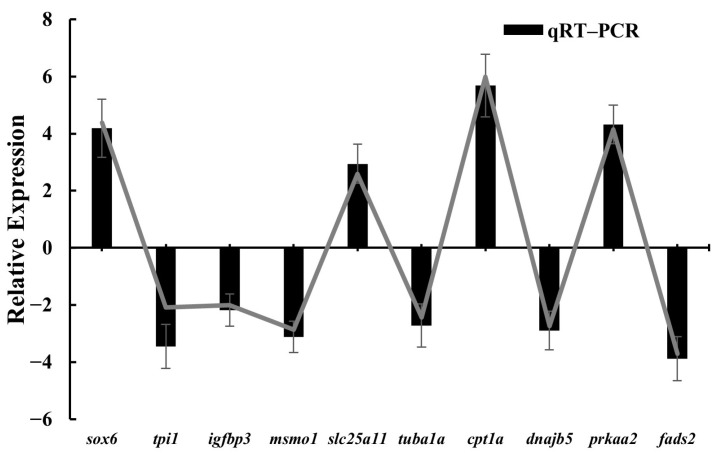
Validation of RNA-Seq results by qRT-PCR in bighead carp orbital fat across two developmental stages. Gene expression levels are presented as mean normalized ratios ± standard error (SE) (*n* = 3 biological replicates).

**Figure A1:**
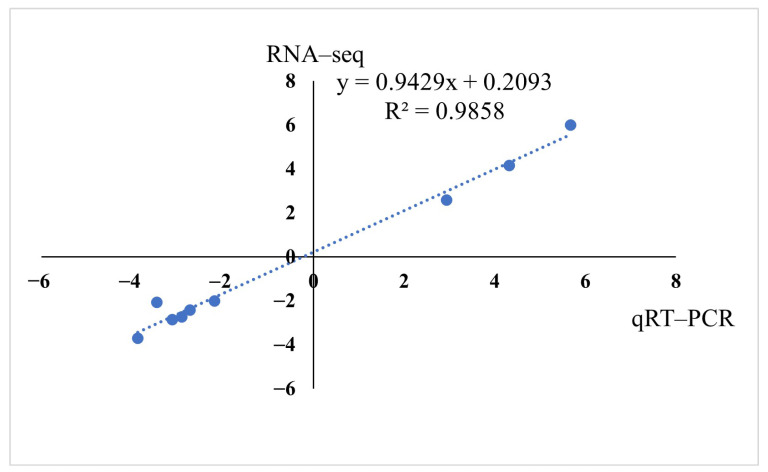
The Linear regression analysis between RNA-seq and qRT-PCR.

**Figure A2:**
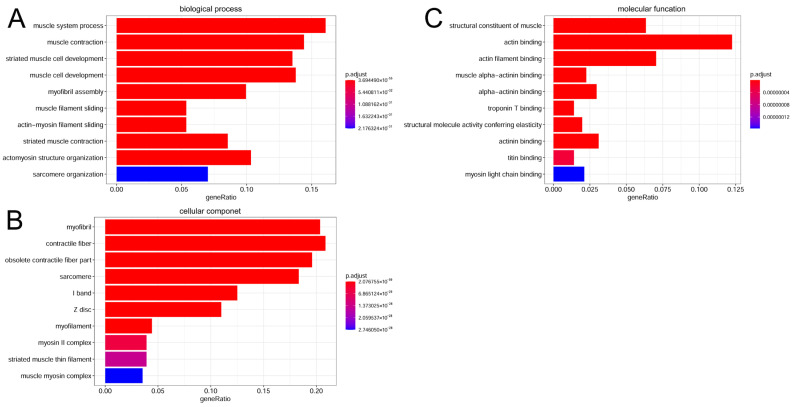
GO enrichment analysis of bighead carp orbital fat transcriptome. (**A**): Biological Process; (**B**): Cellular Component; (**C**) (Molecular Function). Gene ratio (x-axis) reflecting DEG proportion and color indicating *p*-adjust significance.

To verify the accuracy of the RNA-seq data, we randomly selected 10 DEGs for qRT-PC. These randomly selected DEGs included: *tpi1*, insulin like growth factor binding protein 3 (*igfbp3*), methylsterol monooxygenase 1 (*msmo1*), solute carrier family 25 member 11 (*slc25a11*), *tuba1a*, carnitine palmitoyltransferase 1A (*cpt1a*), *dnajb5*, protein kinase AMP-activated catalytic subunit alpha 2; *prkaa2*), SRY-box transcription factor 6 (SRY-box transcription factor 6; *sox6*), and *fads2*. The expression patterns of these 10 DEGs, as determined by qRT-PCR, were consistent with the RNA-seq data (Figure 6). A strong correlation was observed between the two methods (R^2^ = 0.9858, slope = 0.9429; Figure A1), further confirming the high accuracy and reliability of our transcriptome analysis.

## 4. Discussion

Adipose tissue, liver, and skeletal muscle are pivotal organs intimately involved in whole-body lipid metabolism [19,20]. Specifically, adipose tissue functions not only as a metabolic and endocrine organ that mediates *de novo* fatty acid synthesis and lipid storage but also plays an essential role in the systemic regulation of lipid metabolism [21]. As a highly dynamic tissue, it secretes adipokines that contribute to metabolic homeostasis and influences developmental processes. With growing consumer emphasis on food quality, enhancing meat attributes—including texture, flavor, and nutritional profile—has become a critical objective across livestock, poultry, and aquaculture industries [16]. In the present study, we employed high-throughput transcriptomics to elucidate the molecular mechanisms underlying age-dependent alterations in orbital adipose tissue development in bighead carp (*Hypophthalmichthys nobilis*) in vivo. We demonstrate that changes in orbital fat deposition during growth are closely associated with stage-specific regulation of gene expression, providing novel insights into the molecular basis of lipid accumulation in this economically valuable tissue.

In the present study, we obtained some pathways related to lipid anabolism and up-regulated genes in M6 group. Carnitine palmitoyl transferase 1 (*cpt1*) inhibition improves skeletal muscle glucose tolerance and insulin sensitivity. *cpt1b*-/- mice have hearts that are four times larger than controls [22]. The identification of the differentially expressed gene *cpt1b* in the liver transcriptomic analysis of blunt-snout bream (*Megalobrama amblycephala*) individuals, which showed high expression in fast-growing individuals, suggests its potential role in promoting growth by regulating lipid absorption and utilization [23]. High expression of *cpt1b* is positively correlated with growth rate, suggesting that it promotes fish growth through lipid metabolism regulation. Fatty acid transporter protein 1 (*fatp1*), also known as *slc27a1*, is an integral membrane protein that facilitates the influx of long-chain fatty acids and is involved in the genetic network of oleic acid synthesis in beef [24]. Down-regulation of the genes related to lipolytic metabolism in the PPAR signaling pathway, *slc27a1*, *lpl*, *abca1*, and *cpt1a*, has been associated with higher IMF (Intramuscular Fat) in the Wenchang chicken content. The low expression of *slc27a1* enhances chicken intramuscular fat deposition by down-regulating cpt1a-mediated fatty acid oxidation [25]. Furthermore, *slc27a1* was differentially expressed in muscle transcriptome analyses of *Sinocyclocheilus grahami* individuals exhibiting growth differences, which is associated with variations in the efficiency of fatty acid utilization among individuals [26]. In this study, *cpt1b*, *cpt1a* and *slc27a1* genes were all expressed higher in M6 than M18 (Figure 4). It can be hypothesized that the high expression of *cpt1a, cpt1b* and *slc27a1* at M6 may reflect higher fatty acid oxidative capacity or translocation requirements (to support energy demands during the rapid growth period), whereas the down-regulation of these genes at M18 is accompanied by the up-regulation of synthetic genes (e.g., *scd*, *fads2*), which are more favorable for fat deposition.

The high expression of *stk11*, *atp1a2*, *actn2*, and *fbp2* in the M6 group suggests a potential role in suppressing cell proliferation within the orbital adipose tissue. Specifically, *stk11* encodes a serine/threonine kinase involved in cellular polarity and tumor suppression [27], while *atp1a2* has been shown to inhibit tumor growth in vivo [28]. Similarly, overexpression of an ACTN2 fragment suppresses tumor cell motility and proliferation [29]. *prkaa2* encodes a subunit of AMP-activated protein kinase (AMPK), which plays a critical role in various energy metabolism processes. This kinase is implicated in fatty acid biosynthesis pathways and participates in the regulation of energy homeostasis.

High expression of *stk11, atp1a2*, *actn2*, and *fbp2* in the M6 group may inhibit cell proliferation in the adipose tissue of the eye socket, and these genes may be candidate genes associated with bighead head development. In a mice study, it has been demonstrated that lipocalin serves as an important adipose-specific protein, and Ryr3 plays a significant role in the regulation of lipocalin expression. Ryr3 was found to be expressed in 3T3-L1 preadipocytes, and its expression decreased during adipogenesis [30]. The roles of *prkaa2* in energy metabolism have been established in studies on lipid metabolism in the liver of grass carp [31] and in investigations of growth rate variations in *Sinocyclocheilus grahami* [26]. *prkaa2* and *ryr3* expression were lower in the 18-month-old group than in the 6-month-old group, which may be related to the formation of adipose during development. Expansion of adipose tissue (hypertrophy and hyperplasia) is clearly correlated with cell growth and proliferative functions, and studies have reported that the proliferative and differentiation capacity of adipocytes in mice and rats decreases with age [32], but the age of sexual maturity for bighead carp is between 4 and 5 years, and the 6-month and 18-month individuals used for analyses in the present study were at an early stage of their life. Also in this study some genes related to muscle structure, myocyte growth and development regulation such as: *tpm2* and *myl3* genes can regulate muscle growth and muscle fiber differences, while acat1 gene is related to the regulation of lipid deposition capacity and is considered as a candidate gene related to pork quality traits [33]. Ppp2r3a is a major serine/threonine phosphatase with key functions in regulating development and growth [34]. These genes may also be potential candidates for the regulation of bighead carp head traits. Skeletal muscle metabolism and fiber type are related to intramuscular lipid content, and changes in skeletal muscle gene expression affect the development of adipose tissue [35].

We identified 18 genes in the fatty acid metabolism, tight junction, glycolysis/glycolysis and PPAR signaling pathways that were down-regulated in expression in the 6-month-old group. Fatty acid desaturase (FADS2) is a key enzyme in the biosynthesis of polyunsaturated fatty acids (PUFAs), which are the fundamental structural determinants of mammalian membrane lipid bilayers [36]. The gene is involved in fatty acid metabolic processes, arachidonic acid metabolism, RAS, PPAR and VEGF pathways. *fads2* has been positively correlated at the single-cell level with biological behaviors such as tumor inflammation, cell cycle, proliferation, DNA damage, and DNA repair response [37]. The ability of fish to biosynthesis long-chain (≥C-20) polyunsaturated fatty acids varies from species to species, and in teleost fish is largely dependent on the presence of functionally diverse FADS2 enzymes, as many teleosts have lost the gene encoding the Delta 5 desaturase (*fads1*). Nutritional levels may not directly drive diversification of *fads2* in teleost fishes as initially hypothesized, while other factors such as species phylogeny appear to be more influential [38]. Stearoyl coenzyme a desaturase (*scd*) is less expressed in 6-month-olds than in 18-month-olds, and this gene is a key enzyme in the conversion of saturated fatty acids (SFAs) to monounsaturated fatty acids (MUFAs) during lipid biosynthesis [39].

Lipoprotein lipase (LPL) shows differential expression at different stages of the early development of adipose tissue in grass carp [40]. Meanwhile, studies have shown that genes related to adipogenesis may regulate the continuous development of adipose tissue. Existing studies have revealed that the development of white adipose tissue (WAT) in zebrafish is dually regulated by both developmental stage and body size [41]. Adipocytes play a crucial role in metabolism and energy balance. Thus, unlike other organs formed during specific developmental periods, adipogenesis is likely triggered in response to metabolic demands at different growth stages. This may explain the differential expression of lipid-related genes in the orbital fat tissue of bighead carp observed between the two developmental stages in this study. More energy is likely to be focused on the growth of the fish’s skeleton and muscle during the juvenile period at 6 months of age, when growth rates are faster. 18 months of age is the appropriate market size for bighead carp, and the synthesis of PUFAs in the fat of the eye sockets is higher during this period, which is beneficial to the consumer.

In all organisms, carbon fluxes through the fundamental pathways of glycolysis, gluconeogenesis and pyruvate hubs are central processes associated with growth and productivity. Down-regulation of glycolytic enzyme-enolase (*eno1*) expression not only significantly reduces cell proliferation, but also significantly inhibits cell migration, invasion and vivo tumorigenesis [42]. Triosephosphate isomerase 1 (*tpi1*), a key glycolytic enzyme, was identified as a central factor in the glycolytic pathway through an integrated transcriptomics and metabolomics study on feed efficiency in large yellow croaker [43].

Pyruvate kinase L/R (pyruvate kinase L/R; *pklr*) gene is differentially expressed in cows at different lactations and is involved in lipid metabolism through insulin, PI3K-Akt, MAPK, AMPK, mTOR, and PPAR signaling pathways [44]. Overexpression of *graphs* promotes glycolysis, cell growth and proliferation [45]. In this study, we used transcriptome sequencing to compare the major differentially expressed genes in bighead carp orbital fat during different developmental periods, and these energy metabolism-related pathways and genes may also be potential candidate genes for regulating bighead carp growth and development.

## 5. Conclusions

The results showed that several pathways and genes related to cell growth, proliferation and lipid metabolism, including fatty acid metabolism and the PPAR signaling pathway, play important roles in the development of bighead carp eye socket adipose tissues. *cpt1a*, *slc27a1*, *fbp2*, *ryr3*, *fads2*, *scd* and *tpi1* may be the key genes involved in bighead carp fat synthesis and metabolism. Future functional validation studies, such as gene editing, are warranted to confirm the roles of these potential candidate genes. Validated genes could serve as valuable markers for selective breeding programs for growth and meat quality traits in bighead carp future breeding studies.

## Figures and Tables

**Table 1:** Primer sequences of the DEGs for qRT-PCR.

Primers	Sequence (5′-3′)	Tm (°C)
Hyn-tpi1-qpcr-1F	CGGAATCACGGAGAAAGTTGT	59
Hyn-tpi1-qpcr-1R	AGGCTCATAAGCAAGGACCAC	
Hyn-igfbp3-qPCR-1F	TAAGGACGCCGTTAGAGAGC	55
Hyn-igfbp3-qPCR-1R	AGTTTGGAATGCGGAAGC	
Hyn-msmo1-qpcr-1F	TATTTCAGTTCCTCCCTTTCA	55
Hyn-msmo1-qpcr-1R	GCATGGGAGTCCCAGTCATAG	
Hyn-slc25a11-qPCR-1F	AGGCGAGTCACCTTGCTACA	58
Hyn-slc25a11-qPCR-1R	AATGGATTTGGGGCGAAGTTTTT	
Hyn-tuba1a-qPCR-1F	CGTGGTGCCCAAAGATGTG	60
Hyn-tuba1a-qPCR-1R	GGGAGGCTGGTAGTTGATGC	
Hyn-cpt1a-qPCR-1F	TTTTACGACGGACGGTTGC	58
Hyn-cpt1a-qPCR-1R	CTGCTTGTTCTTCCCACGAC	
Hyn-dnajb5-qPCR-1F	CTACGATGTTCTGACCGACCC	59
Hyn-dnajb5-qPCR-1R	CCACCCTTGTTACGGCTGA	
Hyn-prkaa2-qPCR-1F	ACAGCCCTAAGGCACGATG	58
Hyn-prkaa2-qPCR-1R	ACGGGTTCACCACTTTCCA	
Hyn-sox6-qPCR-1F	ACGGAGGTGAGGATGGATT	56
Hyn-sox6-qPCR-1R	GGAGGTTTGTTGTGGAGCA	
Hyn-fads2-qPCR-1F	AGCACGACTTCGGTCATCTATC	58
Hyn-fads2-qPCR-1R	GCACAGTTCCACTACAAACG	
Hynβ-actin-qPCR-F	TATCCTATTGAGCACGGTATTG	57
Hynβ-actin-qPCR-R	CCTGTTGGCTTTGGGATTC	

**Table 2:** Top KEGG pathways and DEGs associated with growth and development of orbital fat of bighead carp.

Regulation	Name of Pathway	Pathway ID	Genes
up	Fatty acid metabolism	ko01212	*hadh*, *cpt1b*, *cpt1a*, *acsl1*
	Tight junction	ko04530	*actn2*, *actn3*, *prkaa2*, *stk11*
	PPAR signaling pathway	ko03320	*slc27a1*, *sorbs1*, *adipoq*
	Glycolysis/Gluconeogenesis	ko00010	*fbp2*, *gapdh*, *aldoa*, *pgm5*
	Cardiac muscle contraction	ko04260	*ryr3*, *atp1a2*, *actc1*, *myh1*, *myl3*
	Adrenergic signaling in cardiomyocytes	ko04261	*ppp2r3a*, *tpm2*, *cacna1s*
down	Fatty acid metabolism	ko01212	*hadha*, *hadhb*, *fads2*
	Tight junction	ko04530	*tuba1a*, *tuba1c*, *cldn7*
	PPAR signaling pathway	ko03320	*scd*, *apoa1*, *lpl*
	Glycolysis/Gluconeogenesis	ko00010	*fbp1*, *hk2*, *eno1*, *eno4*, *gapdhs*, *tpi1*, *pklr*, *ldhb*, *aldoc*

## Data Availability

All sequencing data were uploaded to the Sequence Reading Archive (SRA) of the Nation-al Center for Biotechnology Information (NCBI; accession number: PRJNA1284284).

## Appendix A

**Table A1:** The periocular fat transcriptome data quality control results.

Sample ID	Raw Database	Clean Database	Q20 (%)	Q30 (%)	GC (%)
M6-1	6,871,532,810	6,846,104,587	96.87	91.91	46.97
M6-2	7,485,026,392	7,468,839,798	96.80	91.73	47.73
M6-3	7,616,530,578	7,563,379,410	96.79	91.79	46.35
M18-1	6,431,992,260	6,382,998,824	96.53	91.19	45.37
M18-2	6,100,950,998	6,090,612,188	97.03	92.31	48.38
M18-3	5,914,123,710	5,903,172,099	96.83	91.79	46.60
